# Survival after abdominal impalement with a diver’s harpoon

**DOI:** 10.1016/j.ijscr.2020.02.064

**Published:** 2020-03-07

**Authors:** Abdellatif DJELTI, Hamida JNEID

**Affiliations:** a Department of Orthopaedics and Traumatology, C.H.U. Lamine Debaghine, Bab El Oued, Algiers, Algeria; b Department of General Surgery, C.H.U. Lamine Debaghine, Bab El Oued, Algiers, Algeria

**Keywords:** Abdominal impalement, Transfixing injury, Harpoon gun, Survival, Hepatic injury

## Abstract

**Introduction:**

Abdominal impalement by spear is fortunately exceptional. In the literature, only twenty cases have been so far reported. In most cases, transfixing wounds result in serious visceral and/or vascular lesions with a high risk of mortality. Those cases need careful planning and a preoperative order to properly control the spring harpoon during extraction. The extraction of the spear is a delicate process requiring a thorough study of each case to avoid further damage to the patient. This case is written following the SCARE scale for case report writing.

**Presentation of case:**

We report the case of a 39-year patient, admitted to the hospital only after 13 h after the accident. No visceral or vascular lesion was observed, except minimal liver damage to the 3rd liver segment. The liver was almost spared in only the third segment was lightly touched and regarding the vascular and visceral apparatus, no serious lesions were highlighted.

**Discussion:**

The marking problem we encountered with this patient was that the harpoon ended by a mobile spring beard, which led to a profound lesion and also, to a certain difficulty to get access to it. Also, because of the aorta wound, the surgical approach was chosen, allowing a good exposure of the object.

**Conclusion:**

When it comes to abdominal impalement, following the rules of transporting and mobilizing the patient, using the infectious risk prevention and knowing the importance of the initial assessment of damage aided by radiology, can allow better management of the situation.

## Highlights

• A case of a diver presenting with abdominal impalement by transfixing spear.
• Emergency treatment consisted of surgical exposure and extraction of the harpoon.
• Planification via imaging is primordial to the success of extraction without vital structures idiopathic tears.

## 1 Introduction

Abdominal impalement accidents caused by spears are rarely encountered and treatments are uncommon. They cause serious and often fatal injuries resulting from profound vascular and visceral lesions along with risk of infection. We describe the case of a patient admitted for transfixing abdominal injuries due to impalement with a spear who ultimately survived. This work has been reported in line with the SCARE criteria [7].

## 2 Case presentation

A 39-year-old male patient was admitted to the emergency surgical ward on March 27th 2014 pierced by a harpoon at the abdomen as a result of weapon mishandling. The spear, launched from a distance of 10 cm, pierced the abdomen in the antero-posterior direction. The accident happened 13 h before his admission. At admission, the patient was laying supine right, while he had handled the spear with his both hands. The patient had vomited and fainted several times. He was transported in the supine position according to emergency procedures and was compliant with instructions.

**Fig. 1:**
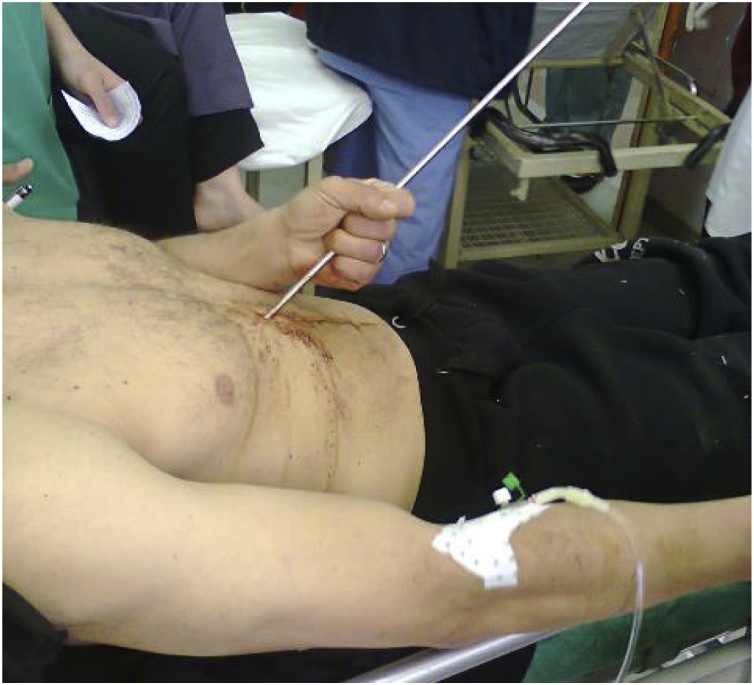
A 39-year-old man victim of an abdominal impalement by a diver's harpoon.

**Fig. 2:**
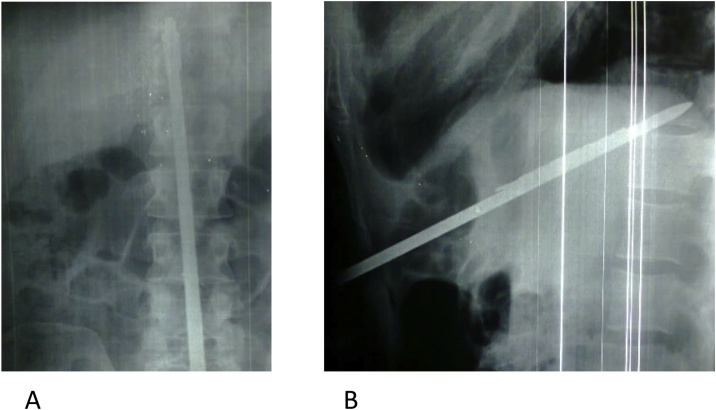
abdominal radiograph (A: face, B: profil) showing the harpoon entry in sub xyphoid, ending in the D11 vertebral body.

**Fig. 3:**
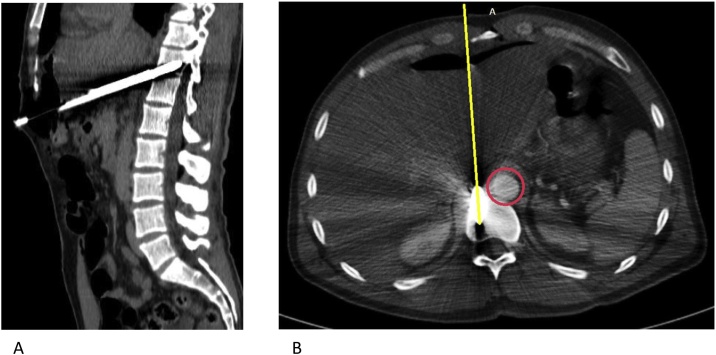
Abdominal CT (A: sagittal, B: frontal) showed the harpoon (yellow line) transfixing the 3rd hepatic segment, crossing the inter aorto-caval space (Aorta in red), and ending at the D11’s vertebral body.

When the patient arrived at the emergency department he was awake and alert, with 120/60 mmHg blood pressure, 86 beats/min heart rate, and 21 cycles/min respiration. Peripheral pulses were all present but some neurological problems were observed. The harpoon, 110 cm long and 1 cm in diameter, had penetrated his abdominal wall at the sub-xiphoid region, making an angle of 70° (Fig. 1). There was slight tenderness and rebounding pain over the epigastric region with no signs of peritoneal syndrome. The patient showed no respiratory distress and his cardiac and breathing sounds did not diminish. Radiographs made in the supine position showed that the harpoon had penetrated at the sub-xiphoid region up to the D11 vertebral body (Fig. 2). Abdominal CT showed the harpoon transfixing the 3rd hepatic segment, crossing the inter aorto-caval space within 4 mm of the abdominal aorta, and ending at the D11 vertebral body (Fig. 3). A small peritoneal effusion was found without any other visceral or vascular lesions. Laboratory tests demonstrated cytolysis syndrome.

The patient was rapidly transported to the operating room after receiving tetanus immunization and intravenous antibiotics, and was managed by a multidisciplinary team comprising visceral and vascular surgeons. Under general anesthesia, the external part of the harpoon was shortened using cutting pliers. We performed a midline sus-umbilical laparotomy bypassing the harpoon. After hepatotomy of the 1st and 3rd hepatic segments, exploration of the omental bursa revealed that the harpoon had shaved the upper edge of the pancreas and penetrated the aorto-caval space through the right diaphragm pillar. After the Kocher maneuver and the dissection of the hepatic pedicle, the harpoon was extracted with peeling of the aorta and manual control of the movable hook of the harpoon. The immediate postoperative course was uneventful. The patient was treated with anticoagulants on the 7th postoperative day due to a portal thrombosis. He was discharged from the hospital one week later.

## 3 Discussion

Impalement injuries are often the result of collisions, suicide attempts, or homicide attempts. These impalements are classified into 3 types [1]: type I lesions result from the impact of a moving body with a stationary object; type II lesions result from the impact of a moving object with a stationary body; type III lesions result from the impact of an object and a body that are both in movement. In our case, the impalement object (a harpoon) is potentially extremely dangerous because the movable hook at its end can rip the viscera and blood vessels if no precaution is used during extraction.

According to the cases described in the literature, victims often benefited from immediate surgical intervention; however, our patient was admitted 13 h after the incident. This was because it was a night-time accident and at the time of the incident he was alone and unable to reach his phone.

Faced with this type of injury, the in-situ object must not be removed in order to preserve its compressive hemostatic effect. However, it can be shortened to facilitate the mobilization of the patient. The patient’s initial position should be maintained to prevent any movement that might aggravate bleeding lesions [2]. All impaling objects must be considered highly contaminated, a good indication for antibiotic and tetanus prophylaxis [[2], [3], [4], [5]]. The position of the patient on the operating table depends upon the size and trajectory of the blunt object, and the type of incision involved [6]. Evaluation of the object’s trajectory is extremely important, because it helps estimate the potential damage [5]. The incision must offer a perfect visual inspection of the object and the adjacent anatomical structures, allowing safe extraction along with no further injuries. In our case, the patient was fortunate that the only observed lesion was a transfixing wound of the 3rd hepatic segment. Immediate and intermediate postoperative monitoring is important to prevent any complications. Our patient presented with a portal thrombosis, which necessitated anticoagulant treatment.

## 4 Conclusion

In cases of abdominal impalement, following the rules of transporting and mobilizing the patient, ensuring infectious risk prevention, and understanding the importance of the initial assessment of damage aided by radiology, can allow better management of the situation. The biggest problem we encountered with this patient was that the harpoon ended with a mobile spring hook which led to a profound lesion and also to difficulty in getting access to it. In addition, due to the aorta wound the surgical approach was chosen, allowing a good exposure of the object.

## Declaration of Competing Interest

No potential conflicts of interest to be declared.

## Sources of funding

No funding source. A case report done by the only author after getting patient consent.

## Ethical approval

The study is exempt from ethnical approval in your institution please state this.

## Consent

Written informed consent was obtained from the patient for publication of this case report and accompanying images.

## Author’s contribution

Dr Abdellatif DJELTI. The only author for this case report.

## Registration of research studies

It is a case report, not a research study.

## Guarantor

Dr Abdellatif DJELTI.

## Provenance and peer review

Not commissioned, externally peer-reviewed.
